# Roles of alternative splicing in modulating transcriptional regulation

**DOI:** 10.1186/s12918-017-0465-6

**Published:** 2017-10-03

**Authors:** Jin Li, Yang Wang, Xi Rao, Yue Wang, Weixing Feng, Hong Liang, Yunlong Liu

**Affiliations:** 1 College of Automation, Harbin Engineering University, Harbin, Heilongjiang 150001 China; 2 Department of Medical and Molecular Genetics, Indiana University School of Medicine, Indianapolis, IN 46202 USA; 3 Center for Computational Biology and Bioinformatics, Indiana University School of Medicine, Indianapolis, IN 46202 USA; 4 Center for Medical Genomics, Indiana University School of Medicine, Indianapolis, IN 46202 USA

**Keywords:** Kidney cancer, Alternative splicing, Transcriptional regulation, Linear regression, GR, MDM2, TP53

## Abstract

**Background:**

The ability of a transcription factor to regulate its targets is modulated by a variety of genetic and epigenetic mechanisms. Alternative splicing can modulate gene function by adding or removing certain protein domains, and therefore affect the activity of protein. Reverse engineering of gene regulatory networks using gene expression profiles has proven valuable in dissecting the logical relationships among multiple proteins during the transcriptional regulation. However, it is unclear whether alternative splicing of certain proteins affects the activity of other transcription factors.

**Results:**

In order to investigate the roles of alternative splicing during transcriptional regulation, we constructed a statistical model to infer whether the alternative splicing events of modulator proteins can affect the ability of key transcription factors in regulating the expression levels of their transcriptional targets. We tested our strategy in KIRC (Kidney Renal Clear Cell Carcinoma) using the RNA-seq data downloaded from TCGA (the Cancer Genomic Atlas). We identified 828of modulation relationships between the splicing levels of modulator proteins and activity levels of transcription factors. For instance, we found that the activity levels of GR (glucocorticoid receptor) protein, a key transcription factor in kidney, can be influenced by the splicing status of multiple proteins, including TP53, MDM2 (mouse double minute 2 homolog), RBM14 (RNA-binding protein 14) and SLK (STE20 like kinase). The influenced GR-targets are enriched by key cancer-related pathways, including p53 signaling pathway, TR/RXR activation, CAR/RXR activation, G1/S checkpoint regulation pathway, and G2/M DNA damage checkpoint regulation pathway.

**Conclusions:**

Our analysis suggests, for the first time, that exon inclusion levels of certain regulatory proteins can affect the activities of many transcription factors. Such analysis can potentially unravel a novel mechanism of how splicing variation influences the cellular function and provide important insights for how dysregulation of splicing outcome can lead to various diseases.

**Electronic supplementary material:**

The online version of this article (doi:10.1186/s12918-017-0465-6) contains supplementary material, which is available to authorized users.

## Background

Regulation of gene expression is one of the most important biological processes in cellular systems. Previously studies suggest that transcriptional regulation can be affected by many factors, such as post-translational modifications of transcription factors, and competitive binding of multiple Transcription factor (TF) [1, 2]. It has been observed that alternative splicing could regulate gene function by adding or removing protein domains, affecting protein activity, or altering the stability of the transcript of the resulting protein [3–6]. However, the role of alternative splicing on the modulations of transcriptional regulation has not been systematically investigated.

Alternative splicing is a critical step of gene regulation and it enables individual genes to generate multiple protein products with different structures and functions by the insertion or deletion of important functional domains encoded by alternatively spliced exons [7]. Differences in the gene expression levels of splicing regulatory factor have been observed in many cancers, and these proteins often affect the splicing patterns of many genes that function in certain cancer-specific biological pathways, including cell cycle progression, cellular proliferation and migration, and RNA processing [8–10]. Although alternative splicing is one of the most widespread mechanisms involved in gene regulation, their roles of acting as modulators to regulate the activity of transcription factor have not been explored.

**Fig. 1:**
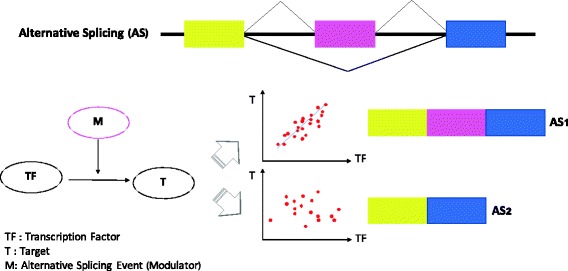
Splicing-centric modulation relationship. The ability of one transcription factor (TF) regulating the expression levels of its target genes (T) is influenced by the percentage of inclusion (PSI) of certain alternatively spliced exons of the modulator protein (M)

As demonstrated in Fig. 1, splicing-centric modulation relationship is defined as the ability of one transcription factor (TF) regulating the expression levels of its target genes (T) is influenced by the percentage of inclusion (PSI) of certain alternatively spliced exons of the modulator protein (M). One example is that when the PSI value of a specific exon in M is high, the expression levels of the putative transcription factor correlates with its targets, while such correlation relation is lost when the PSI value is low.

In this study, we develop a simple regression-based statistical model for evaluating whether the interactions of the percentage of inclusion level of a putative modulator protein and the expression levels of transcription factors jointly contribute to the expression levels of the target genes. A significant interaction term indicates a potentially functional modulation relationship. Tested in KIRC (Kidney Renal Clear Cell Carcinoma) data in the TCGA database [11], our model has the power to identify hundreds of statistically significant modulation relationships using RNA-seq data from only a moderate number of samples. Further investigation suggested that the activity of GR (glucocorticoid receptor) protein has been influenced by the splicing outcomes of p53 and MDM2. This result suggested a potential novel mechanism of how these two proteins influenced cell proliferation and growth in cancer.

## Results

### Model framework

**Fig. 2:**
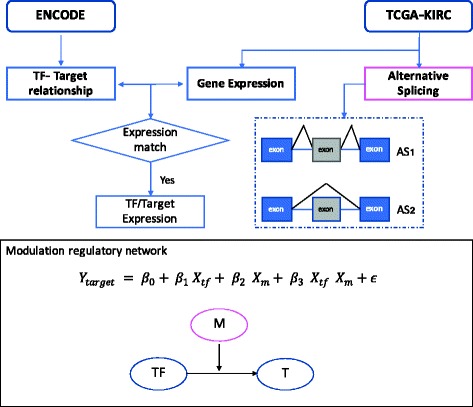
Hypothesis and statistical model. This diagram describes the workflow of data analysis. In the equation portion, X_tf_ and X_target_ represent that the gene expression level of transcription factor and its target, X_m_is the percentage inclusion of a splicing event, *p* value <0.01 indicates the relationship between TF and its target would be influenced by specific modulator

In this study, we construct a regression-based linear model to infer the interactions between the activity of transcription factor (TF), evaluated by correlation between the expression levels of the TF and its targets (T), and the percentage of inclusion of a putative exon in a modulator protein (M). A schematic diagram of workflow is provided in Fig. 2, using the RNA-seq data of the 479 KIRC samples from the TCGA database [11], and the TF-target relationships, derived from the ENCODE (The Encyclopedia of DNA Elements) database. Briefly, the gene expression levels of transcription factors (TF) and their targets (T) were downloaded from the TCGA data portal, which provides the 18,802 values for 20,531 genes. The percentage of inclusion (PSI) of 165 exons was directly derived from.bam files of the RNA-seq data, using a probabilistic model called Mixture of Isoforms (MISO) [12]. These 165 exons were selected from 42,485 annotated skipped exons that are derived using the gene structures of ENSEMBL database, for their correlations with the overall survival outcomes. A complete list of the 165 exons is included in the supplementary information (Additional file 1: Table S1). The splicing outcomes of these 165 exons are considered putative modulators.

In addition to RNA level datasets, the relationships between TFs and their targets were downloaded from the factorbook table, disseminated from the ENCODE database [13]. We focus our analysis on transcription factors and putative targets that with the expression samples over 400. This analysis obtained 226,025 TF-target pairs composed of 83 TFs and 15,597 targets.

We developed a regression-based linear model in deriving the relationship between the activity levels of a transcription factor and the inclusion ratios of an exon in a modulator protein. One equation is formed for describing the relationships among a triplet, [T, TF, M], denotes the expression level of a TF target, the expression level of the TF, and the percentage of inclusion (PSI) of an exon in the modulator, respectively.

We can estimate the relationship as follows:

$$ {Y}_{target}={\beta}_0+{\beta}_1\ {X}_{tf}+{\beta}_2\kern0.5em {X}_m+{\beta}_3\kern0.5em {X}_{tf}\kern0.5em {X}_m+\epsilon $$

where,

- *X*_*tf*_ and *Y*_*target*_ are expression levels of transcription factor and target, respectively,
- *X*_*m*_ is percentage of inclusion of the exon in the candidate modulator protein,
- *β*_1_ and *β*_2_ represent the effect of the TF and the modulator on the expression level of the TF target by themselves alone
- *β*_3_ represents the effect of their interactions.

A positive modulation relationship is equivalent to a non-zero *β*_3_ value, suggesting a statistically significant interaction factors.

### Widespread alternative splicing events modulating transcriptional regulation

**Fig. 3:**
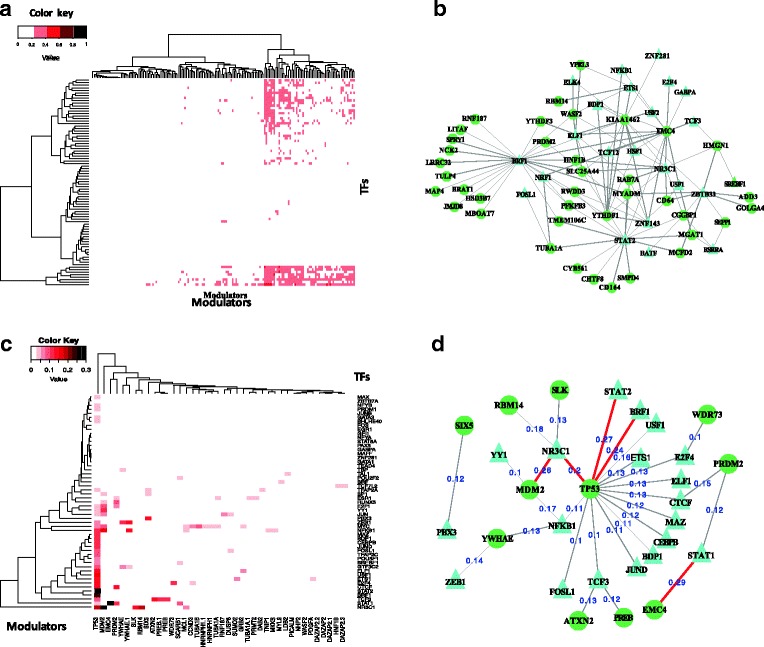
Statistical analysis the relationship between TFs and candidate modulators. Global analysis of TFs and candidate modulators based on the statistical model. **a**. The results of influenced targets percent of 82 TFs via the modulation of 165 differential splicing events. In the heatmap, each row represents a TF and each column indicates a splicing event, the color much darker means a much higher percent targets of TF. **b**. The bipartite network demonstrates the interactions between TFs and candidate modulators which influenced targets percent is more than 30%. Each circle with green color indicates a differential splicing event (modulator), each triangle with blue color represents a TF, the edge between TF and modulator means a physical interaction. **c**. The heatmap shows the percentage of targets of TFs that are influenced by the splicing patterns of modulators. All the TFs and modulators in c should have protein-protein interaction relationships. Each row represents a TF and each column indicates a splicing event. **d**. This network shows the predicted interaction between TFs and modulators which targets percent more the 10%. The red wider indicate target percent over 25%, and all these TFs and modulators here have physically interaction evidence

Based on the model above, we calculated modulation relationships (*β*_3_≠0) among all the 226,025 potential triplets of TFs, targets and candidate modulators. After adjusting for multiple hypotheses correction, we identified 9973 triplets with significant β3 values at FDR (false discovery rate) <0.05 Fig. 3a demonstrated the percentage of targets of individual TFs that are modulated by the splicing outcome of specific modulators. This percentage ranges from 0 to 55.9%. Figure 3b is a bipartite plot that demonstrates the interactions between a candidate modulator (triangle) and a TF (sphere) with more than 30% of its targets influenced. This modulator network is composed of 116 inferred TF-M relationships that include 22 TFs and 40 modulators. In this network, three TFs have relationships with the most number of predicted modulators, including GR, BRF1, and STAT2, with 10, 24, and 17 modulators.

We further examine whether some of the predicted TF-modulator relationships have experimentally-derived interaction relationships. We therefore focused our analysis only on the TF-modulator pairs that have evidence of physical interaction documented in the human protein-protein interaction (PPI) STRING database. Heatmap in Fig. 3c shows the percentage of targets of 53 TFs (rows) that are influenced by the splicing patterns of 40 modulators (columns). In this network, 11,718 TF-modulator pairs were identified with any (>0) targets influenced, and 3137TF-modulator pairs with 10% of TF targets affected. The latter sub-network includes 19 TFs and 11 modulators, whose bipartite relationships are shown in Fig. 3d. Five connections in this network (red line) are composed with TFs with more than 20% of their targets influenced by the modulator protein. These five relationships include 3 modulator proteins and 4 TFs, among which the splicing outcome of TP53 modulates the transcriptional regulation of 3 TFs, including GR, STAT2, and BRF1, and the activation level of GR is influenced by the splicing outcomes of two transcription factors, MDM2 and TP53, two key genes that are involved in the P53 pathways.

### Transcriptional activity of GR is modulated by the splicing outcome of multiple proteins

As shown in Fig. 3b, glucocorticoid receptor (GR) is one of the transcription factors whose transcriptional activity is influenced by the splicing patterns of many modulator proteins. GR is a transcription factor that regulate diverse physiological functions ranging from mitosis to apoptosis. It is essential for embryo maturation, development, metabolism, inflammation, cellular proliferation and survival [14–16]. There are two major mechanisms of gene regulation by GR [17, 18], direct positive regulation via glucocorticoid-response elements (GRE), and indirect regulation that is mediated via crosstalk with other TF proteins, including MDM2 and TP53 [19–21].

**Fig. 4:**
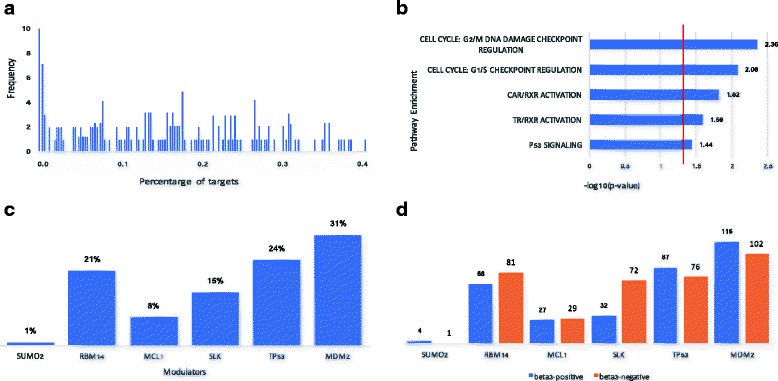
Modulator modulated GR activity. Complete results of GR activity via the modulation of differential modulators. **a** Distribution of GR influenced targets percent via the differential splicing events. **b** Results of Inferred GR modulators enriched canonical pathways. **c** Influenced targets percent via the modulation of 6 different modulators, with exist physical interaction evidence with GR. **d** The number of influenced target genes. The histogram shows the exacted number of genes with FDR < 0.05 based on statistical model we constructed in the study. The *blue* color means β_3_ positive genes, and *yellow* color represents β_3_ negative genes

We systematically evaluate how the splicing patterns of all the putative modulator proteins influenced the GR transcriptional activity. Histogram of the percentage of GR targets that are affected by each of the 165 modulator AS events are shown in Fig. 4a. Among 165 candidate modulator events evaluated, splicing outcomes of 105 events each influences more than 10% of its targets.

After removing of duplicated gene symbols and unannotated genes, 102 genes are associated with theses 105 alternative splicing events, among which, 81 genes can be mapped to the Ingenuity Knowledge Base that are subject to core functional analysis. Canonical pathway analysis suggests that, most of these GR-related modulators are enriched in cell cycle and cell death related pathways, including G2/M DNA damage checkpoint regulation pathway, G1/S checkpoint regulation pathway, CAR/RXR Activation pathway, TR/RXR Activation pathway and p53 Signaling pathway (Fig. 4b).

Based on the human PPI network from STRING database (version 10.0) [22], GR physically interacts with 6 candidate modulator proteins, including SUMO2, RBM14, MCL1, SLK, TP53, and MDM2. The percentage of influenced GR targets ranges from 1% to 31% (Fig. 4c), among which, MDM2 and TP53 influenced the highest percentage of GR-target relationships (31% and 24%, respectively). For each putative modular, the number of targets whose regulatory relationships are positively or negatively influenced differs (Fig. 4d). The positively-influenced targets suggest that more positive (or less negative) relationships between GR and its targets are observed in the samples with higher inclusion levels of the exons in the modular proteins. Similarly, the negatively influenced targets are the targets with more negative (or less positive) relationships with GR in the samples with higher inclusion levels. As shown in Fig. 4d, expression levels of 218 targets were influenced through the modulation of the splicing patterns of MDM2, including 87 and 76 positive-response negative-response targets, respectively. TP53 affects the regulation of 163 GR-targets in total, which is composed of 116 and 102 positively and negatively influenced targets, respectively.

### Splicing outcome of MDM2 protein modulates GR activity

**Fig. 5:**
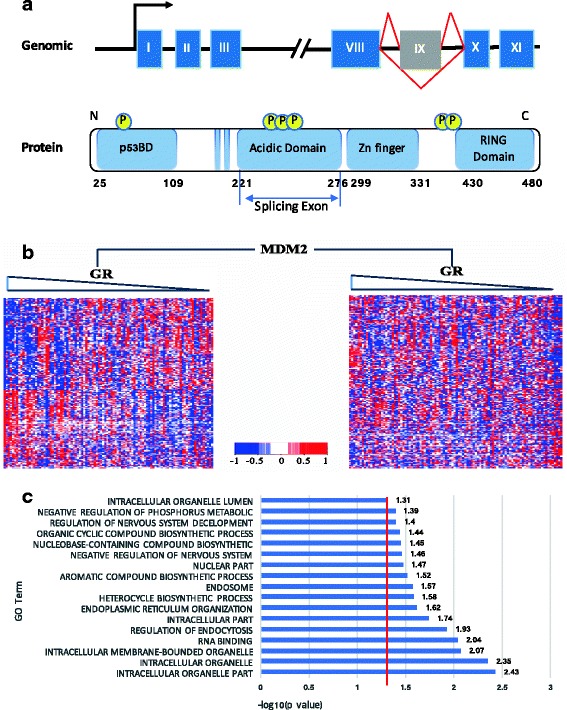
MDM2 as a modulator influence the activity of GR. **a** Genomic and protein structure of MDM2. **b** The heatmap shows the expression level of GR and its targets via the modulation of MDM2. Each row represents a targets, each column indicates a patient, and each row is ordered by the expression level of GR from high to low. **c** The result of GO enrichment of GR targets that influenced by the modulation of MDM2

Inclusion ratio of the 9th exon in MDM2 protein influenced the effects of GR on 31% of its targets. Containing multiple isoforms in both tumors and normal tissues, it is the principal cellular antagonist of the p53 tumor suppresser gene, and inhibits p53 trans-activity by forming a tight complex with p53 [23]. As documented in the UCSC database and described in previously studies, human MDM2 protein is composed of 497 amino acids with eleven exons, and can be divided into four functional-domains, including p53 binding domain, Acidic domain, Zn finger domain and RING domain (Fig. 5a). The alternatively spliced exon is the IX exon, which including L5 protein binding within the central acidic domain (residues 221–276). Previously study reported that GR and MDM2 physically interaction to regulated other proteins or transcription factors. If the splicing event of MDM2 without Acidic domain, it may effect lots of genes binding performance, hence effect their activities with GR.

In order to visualize how inclusion ratios of the alternatively spliced exon (IX exon in MDM2) influenced the relationship between TF and its target, we assess the difference in correlation between the expression level of GR and its targets within the groups of samples with higher and lower inclusion ratios on the IX exon, respectively. The two groups of samples were selected based on the percentage of inclusion (PSI) of the IX exon in MDM2. The high and low inclusion group contain samples with top and bottom 30% of PSI values. As shown in Fig. 5b, the expression level of GR showed clear trend of positive or negative correlation in the group of samples with lower inclusion ratio, while such trend disappears in the high inclusion group. This result clearly indicates that the expression level of GR and its targets indeed are affected by splicing outcomes of the MDM2 protein.

We conducted Gene Ontology (GO) Enrichment analysis on the GR targets whose GR-induced expression changes are influenced by the splicing outcome of MDM2 protein. Most of these GR targets are enriched in cellular component and biological process related categories, from biosynthetic process to intracellular part. The result of GO analysis is shown in Fig. 5c, and the top 5 enriched classes are intracellular organelle, intracellular organelle part, intracellular membrane-bounded organelle, RNA binding, and regulation of endocytosis.

In order to further subcategorize how the inclusion ration of the IX exon in MDM2 influenced its activity, the differences in the correlation between the expression levels of GR and its targets within the samples with high or low inclusion ratios were examined.

As summarized in Table 1, splicing outcome of MDM2 totally affect the GR activity on 218 of its targets, with116 and 102 targets positively and negatively influenced, respectively. Among the 116 positively influences targets, 93 targets showed increased correlation with GR, either from no correlation to significantly positive correlation (23 targets) or from negative correlation to no correlation (70 targets). In addition, we observed reduced negative correlation on 6 targets, and transition from significant negative correlation to positive correlation on 12 target genes. Similarly, the 102 negatively-influenced targets, 65 genes transition from positive correlation to no correlation, while 18 genes change from no correlation to significant negative interaction. There are also 7 targets showed positive correlation, and 12 targets changes from significant positive correlation to negative correlation.

**Table 1:** Targets action changed by the modulator of MDM2

Predicted model of action	Counts	Active	Repress	Inverts
Positive	116	Non-sig → + 23	‘-’ → ‘-’ 6	‘-’ → ‘+’ 12
		- → Non-sig 70	‘+’ → ‘+’ 0	
Negative	102	+ → Non-sig 65	‘-’ → ‘-’ 0	‘+’ → ‘-’ 12
		Non-sig → - 18	‘+’ → ‘+’ 7	

MDM2 as modulator affect the relationship between GR and its targets. Among all the GR targets, MDM2 plays a positive modulation role in 116 targets and plays a negative role in 102 targets (*positive and negative were indicated by *β*_3_value)

**Fig. 6:**
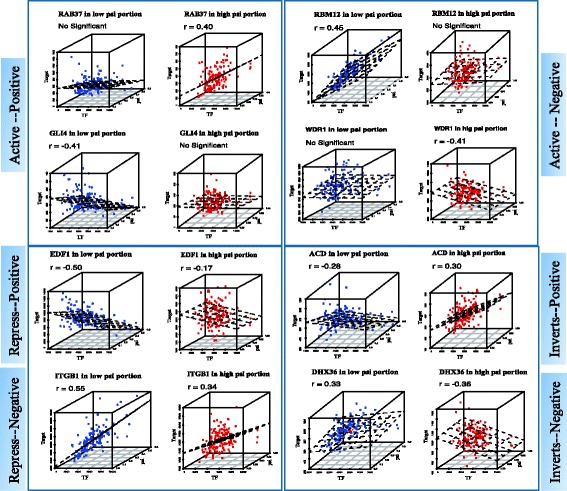
Different modulators influence GR activity. Visualization of the differential correlation between GR and its targets via differential splicing events. These *blue/red* points represent genes are regulated by modulators in low psi portion and high psi portion, respectively. X-ax is expression level of TF, y-ax is expression level of Target, z-ax is psi value of modulator

To further visualize the changes on the correlation levels between GR and its targets with different levels of exon inclusion in MDM2, we randomly selected 8 targets from each category in Table 1, and generated 3D plot demonstrating the relationship among the triplet [TF, T, and M] for samples with low and high PSI values respectively (Fig. 6). For example, no correlation between GR and target WDR1 was observed for the samples with low PSI values on MDM2 gene, as opposed to the positive correlation for the samples with high PSI values.

We conducted Ingenuity pathway analysis (IPA) on positively- and negatively-influenced GR targets, respectively (Table 2). Different sets of enriched pathways were observed for two groups of targets. Cell death and survival, post-translational modification and cellular development pathways are significantly enriched for the positively influenced targets, while pathway related to cell morphology, molecular transport, cell-to-cell signaling and interaction are significantly enriched for the negatively influenced ones. Interestingly, Organismal injury and abnormalities were observed in both two groups of targets, while cancer is only identified in the positively-influenced targets.

**Table 2:** Disease and Bio Functions analysis of influenced targets

Up-regulated targets		Down-regulated targets	
Molecular and Cellular Functions			
Cell Death and Survival	25	Cell Morphology	15
Cell Morphology	7	Molecular Transport	8
Cellular Development	8	Protein Synthesis	3
Cellular Growth and Proliferation	7	Protein Trafficking	2
Post-Translational Modification	16	Cell-To-Cell Signaling and Interaction	7
Diseases and Disorders			
Infectious Disease	6	Developmental Disorder	11
Cancer	101	Hereditary Disorder	21
Organismal Injury and Abnormalities	103	Metabolic Disease	13
Tumor Morphology	3	Organismal Injury and Abnormalities	65
Connective Tissue Disorders	10	Renal and Urological Disease	10

IPA analysis results for GR influenced targets when MDM2 as modulator, the list of top 10 terms of function enrichment and related diseases. (*p*-value <0.01)

### Splicing outcomes of TP53 modulates GR activity

**Fig. 7:**
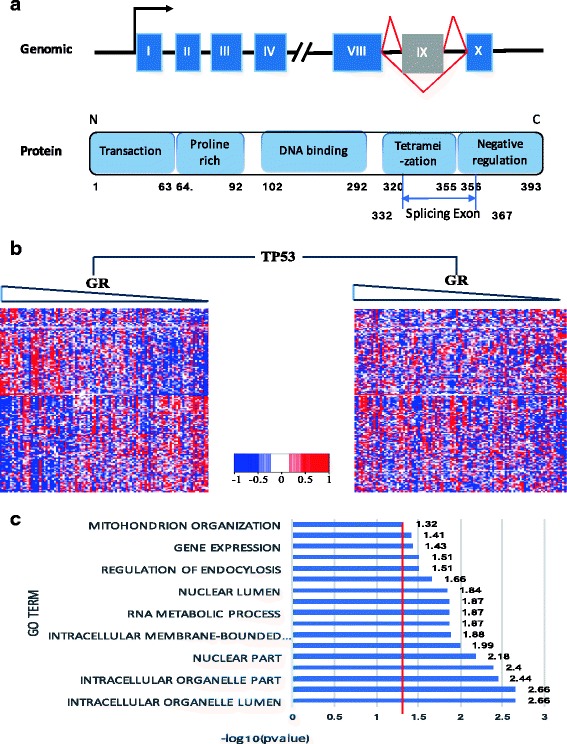
TP53 as a modulator affects the activity of GR. **a** Genomic and protein structure of TP53. **b** The heatmap shows the expression level of GR and its targets via the modulation of TP53. Each row represents a targets, each column indicates a patient, and each row is ordered by the expression level of GR from high to low. **c** The result of GO enrichment of GR targets that influenced by the modulation of TP53

Another key predicted modulator that physical interaction with GR is TP53. TP53 is a protein of 393 amino acids with ten exons and can be divided into five sub-domains, including Transaction domain, Proline rich domain, DNA binding domain, Tetramerization domain and Negative regulation domain (Fig. 7). In this study, we foucs on exon IX ranging from residues 221 to 276, which contains a portion of Teramerization domain and Negative regulation domain.

Figure 6b shows that the relationship between the expression level of GR and its targets differs in the samples with low PSI values in TP53 gene comparing to the sample with high PSI values. Enrichment analysis suggests that 5 GO categories are enriched for the TP53-modulated GR targets, including mitochondrion organization, cellular macromolecule metabolic, spliceosomal complex, cellular macromolecule biosynthetic, and nucleus signaling pathway.

## Discussion

In this study, we proposed a regression-based linear model to infer the interactions between the activity of a transcription factor (TF) and the exon inclusion percentage of a modulator protein (M). Using the RNA-seq data from TCGA KIRC program, we investigated 83 TFs and 165 candidate modulator proteins and identified 116 significant TF-M interactions (total 9973 TF-M-target triplets). Then, we constructed an interaction network using TF-M pairs and further refined the network by only including TF-M pairs that have documented physical interaction from STRING database.

Among the inferred interactions, GR is one of the TFs whose transcriptional activity is affected by multiple modulator proteins, including MDM2 and TP53. The percentage of GR targets influenced by the MDM2 and TP53 is 31% and 24%, respectively. We further examined these interactions in PPI network and found that GR protein does have physical interaction with both MDM2 and TP53, further validating the effectiveness of our model. Meanwhile, pathway analysis shown that many influenced GR targets are enriched in pathways that are associated with cancer, including p53 signaling pathway, G1/S checkpoint regulation pathway, and G2/M DNA damage checkpoint regulation pathway. Previous study reported that endogenous TP53 and GR can form a ligand-depend trimetric complex with MDM2 in the cytoplasm, which may act as opposing forces in the decision between cell death and survival [24]. Our results suggest that differential splicing status of MDM2 and TP53 may play distinctly functions on modulating GR transcriptional activity, leading to different decision between cell death and survival.

Previously, several computational methods have been developed to identify modulators whose expression levels could affect the regulation activity of transcription factors toward its target genes. Wang and Califano et al., [25] proposed an information theoretic algorithm for detecting modulators. They tested the CMI (conditional mutual information) between the expression levels of TF and T, and its dependency on the modulators. Building upon the same principle, Babur et al., [9] presented a probabilistic method for detecting modulators of TF using a priori knowledge and gene expression profiles. Wu et al., [26] proposed an approach to infer ER/modulator/target relationship, where gene expression data and Chip-seq data were used to construct a genomic/non-genomic regulatory networks. All these three studies focus on how the expression levels of modulator proteins affect transcriptional regulation. However, different isoforms of a protein may have different functions. In our study, we systematically investigated the role of alternative splicing in modulating transcriptional regulation and for the first time reported that exon inclusion levels of regulatory proteins can act as modulators and affect the activities of many transcription factors. Such analysis will provide important insights into how dysregulation of RNA splicing can lead to various diseases.

## Conclusions

Our study suggested a novel mechanism of alternative splicing acting as modulators to modulate transcriptional regulation. Using clear cell renal carcinoma as an example, we comprehensive analyzed GR whose interaction with targets could be altered due to differential splicing status of the modulator protein MDM2 and TP53. Based on the results, we demonstrated that this modulation existed generally within the cell, which might be also observed in other cancer types and normal cells. Our finding added another level of transcriptional regulation and raised the potential of alternative splicing as a therapeutic target.

## Methods

### Expression datasets obtain

Paired-end RNA-seq of KIRC including 480 patients was download form TCGA. Using the software mixture-of-isoforms (MISO) to calculate the exon inclusion level (PSI, Percent of Spliced In) of skipped exons, it was obtained from unpublished data of XiRao previously analysis in our lab.

Transcription factors were obtained from UCSC factorbook (http://factorbook.org) [27] and used Chip data from ENCODE project (http://www.genome.gov/Encode/) to predict target genes. We defined the promoter region is upstream/down 1000 bp of a gene. By searching the binding site of a gene promoter region, we obtained the candidate target genes. Filtering those TFs and target genes without expression data in kidney cancer. 165 alternative splicing events as candidate modulators which highly correlation with kidney cancer survival were obtained from XiRao unpublished data.

### Regulation function and preprocessing data

In order to accurate assess the correlation among each triplet, we filtered out those outlier samples beyond the border of *mean* ± 3*std* of each gene (target and transcription factors) expression level. Reset psi value range from [0.01 ~ 0.99] as following:

$$ {X}_m=\left\{\kern0.5em \begin{array}{c}0.01,\kern2.25em {X}_m=0\\ {}{X}_m,\kern1.5em {X}_m\in \left(0,1\right)\\ {}0.99,\kern0.5em {X}_m=1\end{array}\right. $$

Meanwhile, we made a log_2_(*X*_*tf*_ + 1), log_2_(*X*_*target*_ + 1) transformation to the expression level of TF, target, and transform psi value into ${\mathrm{log}}_2\left[\left(\frac{X_m}{1-{X}_m}\right)+1\right]$, respectively.

After transforming our expression data and splicing data, our model becomes:

$$ {Y_{target}}^{\prime }={\beta}_0+{\beta}_1\ {X_{tf}}^{\prime }+{\beta}_2\kern0.50em {X_m}^{\prime }+{\beta}_3\ {X}_{tf}^{\prime }\ {X_m}^{\prime }+{\epsilon}^{\prime } $$

where,

*X*_*tf*_′ and *Y*_*target*_′ are transformed expression levels of transcription factor and target,

*X*_*m*_′ is transformed psi value of candidate splicing modulator,

*ϵ*′ is the error.

Given a set of expression profiles and the psi values of modulate splicing events, we estimate *β*_3_ coefficient by calculating the proportion of target conditional on TF and modulator. We then select triplets with a high *β*_3_ coefficient that satisfy a false discovery rate threshold after multiple hypothesis testing condition. In this model, we assume that the more significantly the *β*_3_ coefficient the more interactions will be affected between TF and targets.

### Database and related software

Statistical analysis and processing of the data were performed using R version 3.0.1 (www.r-project.org). RNA-seq expression data was download from TCGA, and alternative splicing values were calculated by MISO (Mixture of Isoforms) software. DAVID [28, 29] and IPA (Ingenuity pathway analysis) were used to make gene function and pathway analysis. Protein-protein interactions was predicted by STRING database (http://string-db.org) [22]. Transcription factor Chip-seq data was download from ENCODE (the encyclopedia of DNA Elements, http://encodeproject.org) [13].

## Additional file

Additional file 1: Table S1. 165 Alternative Splicing Events whose PSI values show high level of correlation with kidney cancer survival outcome. (XLS 77 kb)

## Abbreviations

CV: Coefficient of variation
DAVID: The database for annotation, visualization and integrated discovery
ENCODE: Encyclopedia of DNA Elements
GO: Gene ontology
GR: Glucocorticoid receptor
IPA: Ingenuity pathway analysis
KIRC: Kidney renal clear cell carcinoma
M: Modulator
MDM2: Mouse double minute 2 homolog
MISO: Mixture of Isoforms
PSI: Percentage spliced in
TCGA: The cancer genome atlas
TF: Transcription factor
TP53: Tumor protein p53
